# Loss of ATG5 impairs CD4+ T cell activation and promotes anti-tumor responses

**DOI:** 10.3389/fimmu.2025.1284391

**Published:** 2025-09-04

**Authors:** Carlos Plaza-Sirvent, Clara Bessen, Alisha W. Bronietzki, Katjana Klages, Marc Schuster, Jochen Huehn, Ingo Schmitz

**Affiliations:** ^1^ Dept. of Molecular Immunology, Ruhr University Bochum, Bochum, Germany; ^2^ Systems-oriented Immunology and Inflammation Research Group, Helmholtz Centre for Infection Research, Braunschweig, Germany; ^3^ Institute of Molecular and Clinical Immunology, Otto-von-Guericke University, Magdeburg, Germany; ^4^ Department of Experimental Immunology, Helmholtz Centre for Infection Research, Braunschweig, Germany

**Keywords:** autophagy, ATG5, CD4 T cell, regulatory T cell, T cell activation

## Abstract

Macroautophagy (hereafter called autophagy) is an ancient catabolic process that delivers bulky cargo to lysosomal degradation. The autophagic pathway is regulated by autophagy-related (ATG) proteins that govern the formation of double-membraned vesicles called autophagosomes. Autophagy has been shown to be important for T cell survival and proliferation. However, all studies performed so far used genetic models, in which deletion of an essential *Atg* gene occurs at early stages of thymic T cell development, raising the question whether developmental defects account for the phenotypes observed in mature T cells. Especially regarding CD4^+^ T helper cells, little is known about the function of autophagy in specific subsets. Therefore, we generated mice that lack *Atg5*, an essential component of the core autophagy machinery, in activated CD4^+^ T cells using OX40-Cre mice. As expected, thymic T cell development was unaffected in these mice. Despite impaired CD4^+^ T cell activation, Atg5^ΔOX40^ mice developed lymphadenopathy and exhibited increased T cell numbers, pointing to a defect in immune regulation. Accordingly, frequencies of Foxp3^+^ regulatory T (Treg) cells were decreased. While activation-induced cell death and *in vitro* suppressive activity of Treg cells were not affected, ATG5 deficiency in CD4^+^ T cells led to increased anti-tumor responses against melanoma. In conclusion, our data suggest that ATG5 is crucial for the functional properties of CD4^+^ T cells and the homeostasis of Treg cells.

## 1 Introduction

Unwanted organelles, protein aggregates, or even intracellular pathogens are degraded via the lysosomal pathway by an ancient catabolic process called autophagy. The bulky cargo to be degraded is initially enclosed in double-membraned vesicles known as autophagosomes (1, 2). The autophagic machinery consists of two ubiquitin-like conjugation systems, in which first the ubiquitin-like molecule ATG12 is conjugated to ATG5 (3). The ATG5–12 complex, together with ATG16L1, associates with nascent autophagosomes and acts as an E3 ligase to conjugate the ubiquitin-like molecule ATG8 to the lipid phosphatidyl-ethanolamine (4). As part of the core autophagic machinery, the ATG8 protein family participates in the elongation of the nascent autophagosome and closure of the double membrane vesicle, which finally fuses with a lysosome to degrade the cargo (5). Despite being a conserved process among species, only one ATG8 protein exists in yeasts, while six different proteins, namely LC3A, LC3B, LC3C, GABARAP, GABARAPL1, and GATE-16, can be found in mammals (3).

CD4^+^ T helper (Th) cells are a central part of adaptive immunity. They orchestrate the adaptive immune response via membrane-bound co-stimulatory ligands and the secretion of soluble cytokines. In order to cope with the challenges of extracellular and intracellular pathogens, they differentiate into various effector subsets such as Th1, Th2, and Th17 cells that tailor the immune response accordingly (6). Besides their effector function, T cells are also fundamental for the regulation of the immune response. A subset of CD4^+^ T cells, characterized by the expression of the transcription factor Foxp3, employs immunosuppressive mechanisms to dampen excessive responses of the immune system (7, 8). Expression of inhibitory receptors like CTLA-4, IL-2 sequestration, or production of inhibitory cytokines can be found among the mechanisms used by Treg cells to suppress the immune system (9, 10). Treg cells play an essential role in the maintenance of immune homeostasis, and a misbalance in the ratio of Treg cells to the effector arm of the immune system causes or favors the rise of pathologies. For instance, excessive Treg cells impair immunosurveillance of tumor cells and can give rise to cancer (11). In fact, tumor cells recruit Treg cells via the Neuropilin1 (Nrp1)-VEGF axis to generate an immunosuppressive environment (12). Contrariwise, defects in the suppressive function of the Treg cells or reduction in their numbers result in autoimmune disease as observed in the human IPEX syndrome and the murine scurfy mutation, both characterized by non-functional Foxp3 (13–15), or upon depletion of Treg cells (16, 17).

T cell development and naïve T cell homeostasis are biological processes that critically rely on autophagy (reviewed in (18, 19)). Previously, we and others have shown the importance of autophagy in the maintenance of the immune homeostasis by Treg cells (20–23). Furthermore, it was demonstrated that autophagy has an essential role in the formation of a protective CD8^+^ memory population (24, 25). Here, we created a novel conditional knockout mouse line to investigate the function of autophagy in activated CD4^+^ T cells. In line with Treg cells having an activated phenotype, we confirm the importance of autophagy for Treg homeostasis, phenotype, and function. Moreover, we show that autophagy is important for the proper activation of conventional CD4^+^ Th cells. Despite impaired CD4^+^ T cell activation, *Atg5* deficiency in activated CD4^+^ T cells led to an enhanced anti-tumor response.

## 2 Materials and methods

### 2.1 Mice

Atg5^fl/fl^ and OX40-Cre mice were described previously (26, 27) and have been kindly provided by Dr. Mizushima and bought from The Jackson Laboratory, respectively. Atg5^ΔOX40^ mice were generated by crossing Atg5^fl/fl^ with OX40-Cre mice. To test for OX40-Cre activity, OX40-Cre mice were crossed with ROSA^mT/mG^ mice [Gt(ROSA)26Sor^tm4(ACTB-tdTomato,-EGFP)Luo^/J], which have been described previously (28). All mice were kept under specific pathogen-free conditions in the animal facility of the Helmholtz Center for Infection Research (HZI), Braunschweig (29). Animal experiments and breeding were performed following the guidelines of local and national authorities.

### 2.2 B16 melanoma model

The B16 tumor model was performed as previously described (30). Briefly, 2 x 10^6^ B16 melanoma cells were subcutaneously injected into the right flank of the mice, and tumor growth was monitored by measuring the orthogonal tumor diameters. Mice were sacrificed if the mean tumor diameter reached 12 mm.

### 2.3 Flow cytometric analyses

For surface marker staining, cells were resuspended in 100 μl FACS buffer (2% BSA in PBS) and incubated in the presence of the respective antibodies for 15 minutes at 4 °C in the dark. Afterwards, cells were washed twice with 500 μl FACS buffer and analyzed using an LSRII flow cytometer (BD Biosciences). Before surface staining, in the case of cell population analyses (excluding viability determinations), dead cells were excluded by incubating the cell suspension with LIVE/DEAD^®^ Fixable Blue Dead Cell Stain staining (LifeTechnologies) for 30 minutes at 4˚C in the dark and subsequently washed twice with PBS; afterwards Fc receptors were blocked by 15 minutes incubation with Fc-block (CD16/32) in FACS buffer at 4˚C and washed with FACS buffer. For intracellular proteins, staining was performed using Foxp3 Staining Buffer Set (Miltenyi, #130-093-142) according to the manufacturer’s instructions. Antibodies used were: CD4-FITC (RM4-5, eBiosciences), CD4-PacificBlue (L3T4, BioLegend), CD8-PECy7 (53-6.7, Biolegend), CD25-PE-Cy7 (PZ61, BD Pharmigen), CD44-APC (IM7, Biolegend), CD62L-PerCPCy5.5 (MEL-14, eBioscience), CD134(OX40)-PE (OX86, Biolegend), CD134(OX40)-APC (OX86, Biolegend), CTLA-4-PE (UC10-4P9, eBioscience), Foxp3-AlexaFluor488 (FJK-16s, eBioscience), GITR-PECy7 (DTA-1, eBioscience), GITR-PE (DTA-1, BD Pharmigen), Helios-PE (22F6, Biolegend) and Nrp1-PE (3E12, Biolegend). Samples were acquired on LSR Fortessa or LSR II (Becton, Dickinson and Company) and analyzed by FlowJo software (Tristar).

### 2.4 Activation-induced cell death assay

Cells isolated from peripheral lymph nodes of *Atg5^ΔOX40^* and *Atg5^fl/fl^* control mice were seeded in primary T cell medium, i.e. RPMI1640 supplemented with 10% FCS (PAA Laboratories), 50 μM β-mercaptoethanol, 50 μg/ml each of penicillin and streptomycin, 1% non-essential amino acids and 1 mM sodium pyruvate (all from Life technologies) and stimulated with Concanavalin A (1.5 μg/ml) and IL-2 (100 ng/ml). After 72 hours, cells were re-stimulated with pre-coated anti-CD3 (10 μg/ml), PMA (100 ng/ml) + Ionomycin (1μg/ml), CD95L (100 ng/ml), or left untreated for additional 24 hours. Subsequently, cells were stained with 7-AAD (Biolegend), CD4, and CD134 antibodies to determine cell viability in a Cytoflex LX (Beckman Coulter) flow cytometer.

### 2.5 *In vitro* Treg suppression assay

CD4+ CD25− conventional T cells (Tcon) and CD4+ CD25+ regulatory T cells (Treg) were isolated from mouse spleen using the mouse CD4+CD25+ Regulatory T Cell Isolation Kit, (#130-091-041, Miltenyi). Tcon cells were stained with Cell Trace Violet according to the manufacturer´s protocol (C34557, ThermoFisherScientific). Afterwards, Tcon and Treg cells were co-cultured in primary T cell medium and stimulated using T Cell Activation/Expansion Kit, mouse (130-093-627, Miltenyi) supplemented with IL-2 (100 ng/ml). Treg: Tcon cells were seeded in the indicated ratios. After 72 hours, cells were harvested and stained with CD4-FITC (RM4-5, eBiosciences) and CD25-PE (Miltenyi) and analyzed for proliferation in a Cytoflex LX (Beckman Coulter).

### 2.6 Immunoblotting

OX40- and OX40+ cells were purified using a CD4+ CD25+ regulatory T cell isolation kit for magnetic separation (130-091-041; Miltenyi Biotec) according to the manufacturer’s protocol, with the modification that the anti-OX40-PE (#119410, BioLegend) was used instead of the anti-CD25-PE provided in the kit. Cells were lysed by incubation in TPNE buffer (1x PBS add 300 mM NaCl, 1 mM EDTA, 1% Triton X-100) supplemented with protease inhibitors for 20 minutes on ice. After centrifugation, protein concentration was determined by BCA assay (ThermoFisher Scientific). Protein lysates were loaded onto 12% SDS-polyacrylamide gels for ATG5 or 15% SDS-polyacrylamide gels for LC3, blotted onto PVDF membrane (GE Healthcare), and detected by chemiluminescence. Antibodies used for Western blotting: ATG5 (12994S, Cell Signaling Technology), LC3A/B (4801S, Cell Signaling Technology), Beta-Actin (66009-1-lg, Proteintech).

### 2.7 Statistical analysis

Statistical analyses were performed by Mann-Whitney, one-way ANOVA (Tukey’s multiple comparison post-test), or two-way ANOVA (Dunnett´s multiple comparison test) tests to determine statistical significance using GraphPad Prism (GraphPad Software Inc.). Standard error of the mean (SEM) is represented as error bars in the graphs.

## 3 Results

### 3.1 Cre recombinase driven by the Tnfrsf4 promoter deletes in activated conventional CD4^+^ T cells and Treg cells

**Figure 1:**
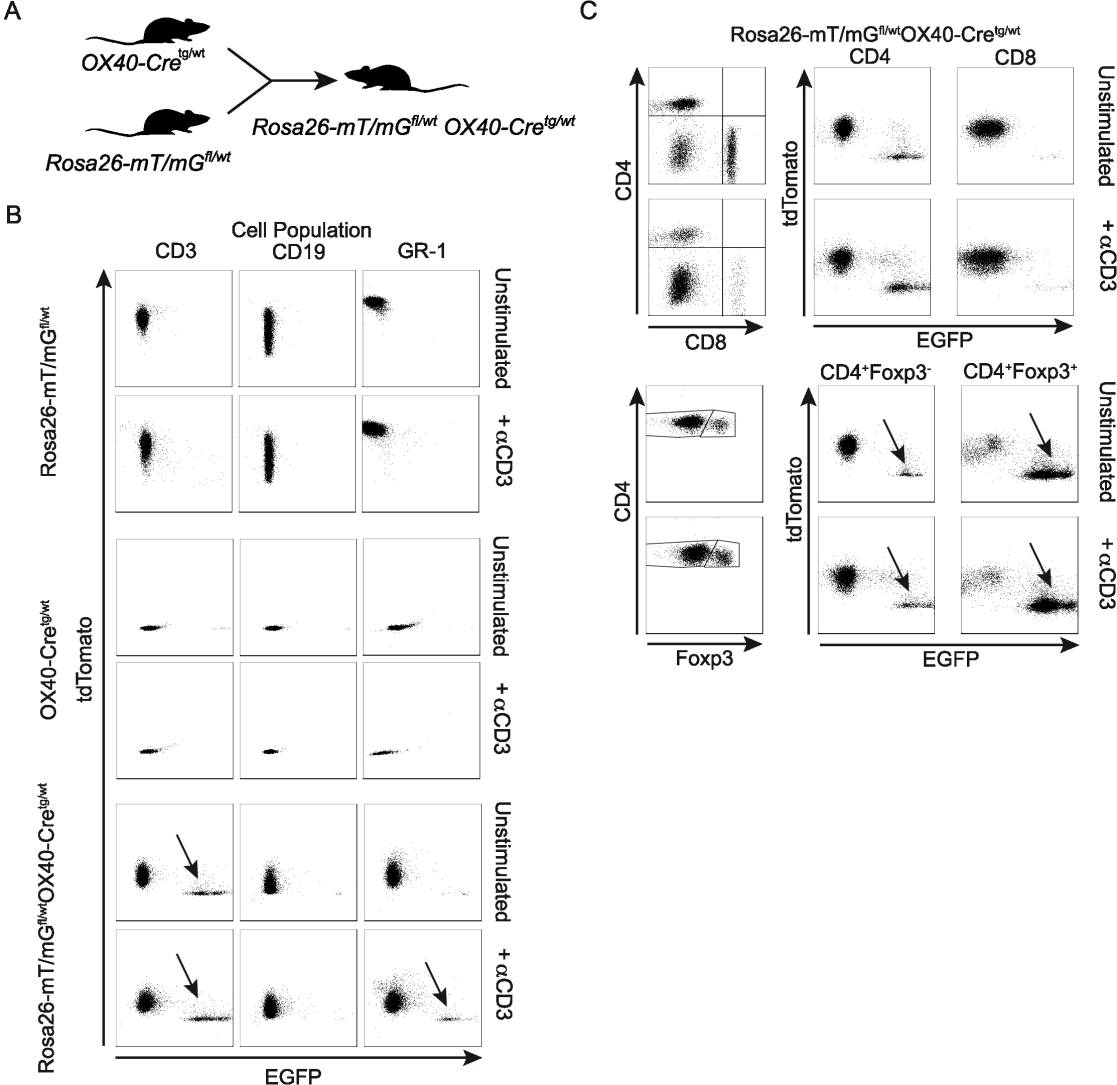
Characterization of the OX40-Cre deleter line. **(A)** Scheme describing the generation of Rosa26-mT/mG^fl/wt^OX40-Cre^tg/wt^ mice. **(B)** Dot plots of tdTomato and EGFP expression within unstimulated or anti-CD3-stimulated CD3^+^, CD19^+^ and GR-1^+^ cells from Rosa26-mT/mG^fl/wt^, OX40-Cre^tg/wt^ and Rosa26-mT/mG^fl/wt^OX40-Cre^tg/wt^ mice. **(C)** Dot plots of tdTomato and EGFP expression within unstimulated or anti-CD3-stimulated CD4^+^, CD8^+^ and CD4^+^Foxp3^+^ cells from Rosa26-mT/mG^fl/wt^OX40-Cre^tg/wt^ mice.

OX40 is a member of the tumor necrosis factor receptor superfamily (TNFRS4, CD134) and is a secondary co-stimulatory molecule that is expressed mainly by activated T cells to prolong their activation and to sustain their proliferation (31). The *Tnfrsf4* promoter has been used to drive Cre expression in activated CD4^+^ T cells and Foxp3^+^ Treg cells (27, 32). Crossing OX40-Cre^tg/wt^ mice with Rosa26-mT/mG^fl/wt^OX40-Cre^tg/wt^ mice (**Figure 1A**), we verified that OX40-driven Cre expression is detectable in CD3^+^ T cells, but not CD19+ B cells (**Figure 1B**). Interestingly, we detected some Cre activity in Gr-1+ cells (**Figure 1B**), which is consistent with the fact that activated Th1 cells express this marker (33). Comparing T cell lineages, OX40-Cre was largely inactive in CD8+ and active in CD4+ T cells, with particularly high recombination efficiency in Foxp3^+^ CD4^+^ T cells (**Figure 1C**).

### 3.2 Autophagy-deficiency in Atg5^ΔOX40^ mice affects mostly CD3^+^ T cells and does not perturb thymocyte development

**Figure 2:**
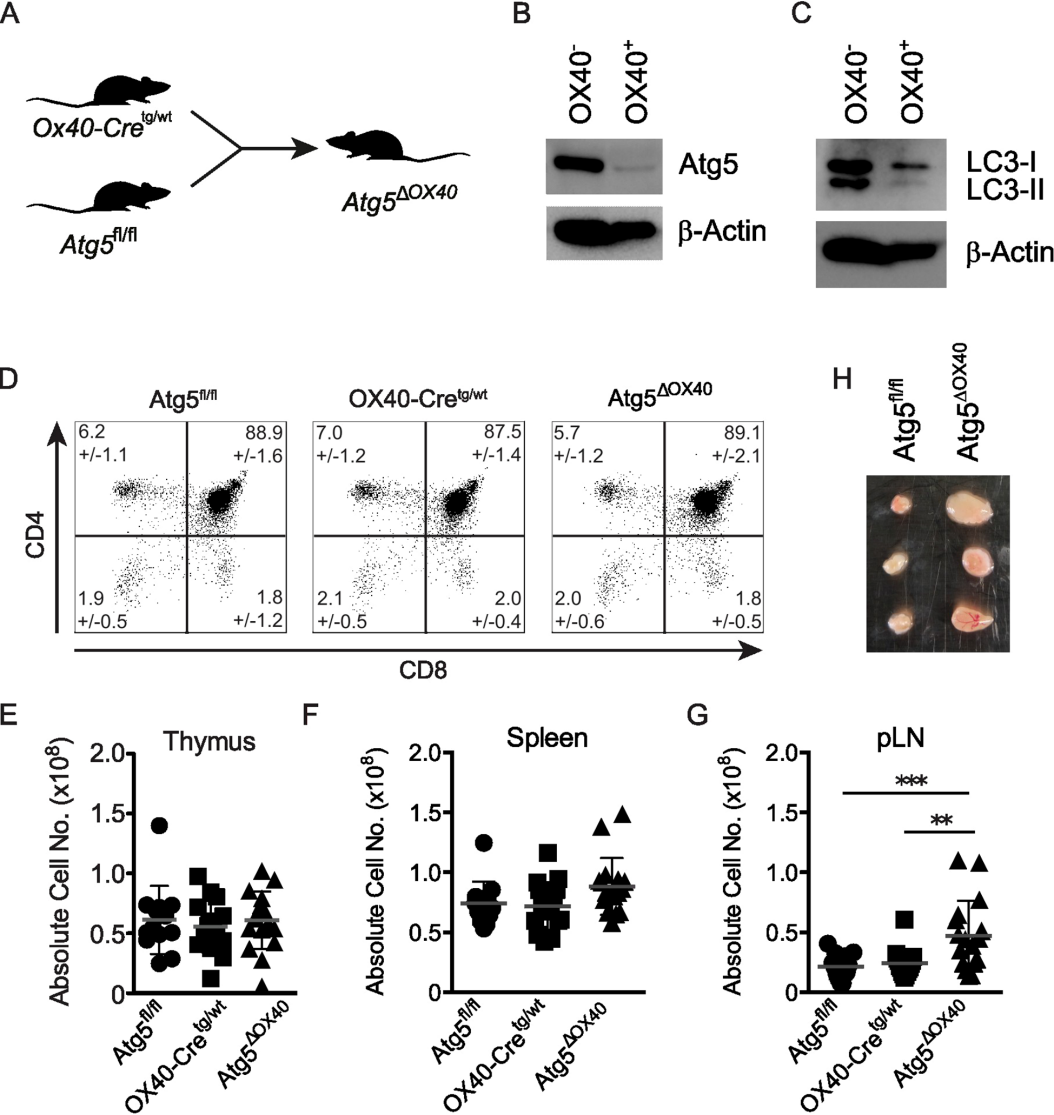
No gross alterations of T cell development in Atg5^ΔOX40^ mice. **(A)** Scheme describing the generation of Atg5^ΔOX40^ mice. **(B, C)** Immunoblot of ATG5 and LC3 expression in OX40-negative and OX40-positive cells from Atg5^ΔOX40^ mice. Actin was used as a loading control. **(D)** Dot plots of thymic CD4- and CD8-expressing cells in Atg5^fl/fl^, OX40-Cre and Atg5^ΔOX40^ mice. Mean frequency +/-SD are given; n=10-12. The one-way ANOVA and Tukey’s multiple comparison tests were used for statistical analysis. **(E-G)** Absolute cell numbers of thymi **(E)**, spleen **(F)** and pLN **(G)** from the indicated mice. Mean and SD are shown; n=12-16. **(H)** Representative examples of lymph nodes from Atg5^ΔOX40^ and control mice. **p < 0.01 and ***p < 0.001.

To investigate the role of autophagy in activated CD4^+^ T cells, OX40-Cre^tg/wt^ mice were bred with Atg5^fl/fl^ mice (26) to generate Atg5^ΔOX40^ mice (**Figure 2A**). Deletion of ATG5 in OX40-expressing CD4+ T cells was confirmed by immunoblotting (**Figure 2B**). In line with ATG5 deficiency, LC3 conjugation to phosphatidylethanolamine was strongly impaired as LC3-I but hardly any LC3-II was detected in OX40-positive CD4+ T cells (**Figure 2C**). Consistent with the lack of Cre activity in the thymus, T cell development was not affected in Atg5^ΔOX40^ mice since both the frequencies of thymocyte populations (**Figure 2D**) and the thymic cellularity (**Figure 2E**) were comparable to control mice. While the cellularity was only marginally affected in the spleen, the effect of autophagy-deficiency in OX40-expressing cells of Atg5^ΔOX40^ mice resulted in a significant expansion of cell numbers in peripheral lymph nodes (pLN) (**Figures 2F, G**). Although not statistically significant, there was a tendency towards a higher cellularity in mesenteric LN (mLN) (data not shown). Accordingly, the lymph nodes of Atg5^ΔOX40^ mice were enlarged, consistent with lymphadenopathy (**Figure 2H**).

**Figure 3:**
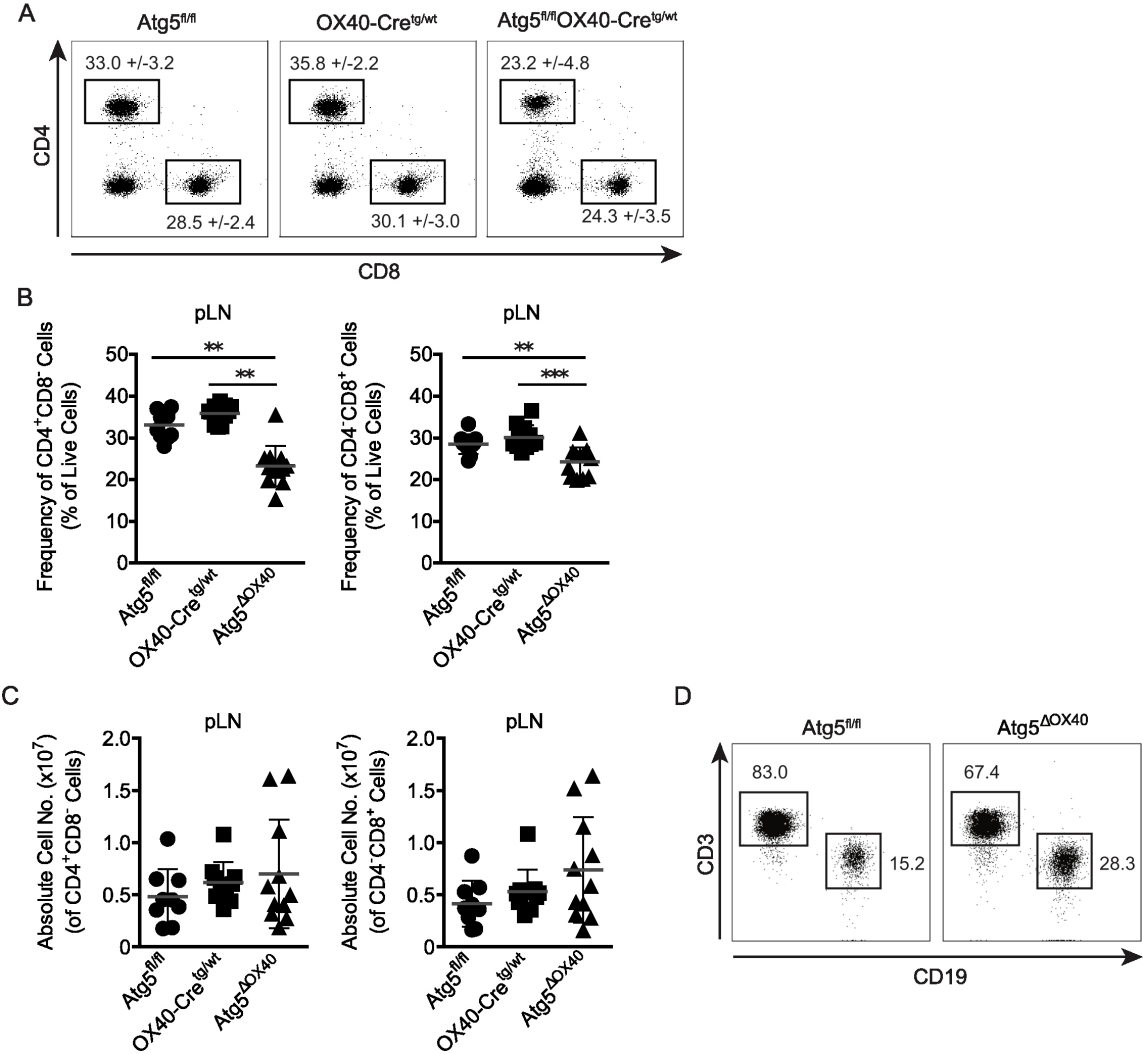
Characterization of the CD4+ and CD8+ T cell compartment in Atg5^ΔOX40^ mice. **(A)** Dot plots of CD4^+^ and CD8^+^ cell populations from pLN of the indicated mice. **(B)** Graphical presentation of frequencies of CD4^+^ and CD8^+^ cells. Mean frequency +/-SD are given; n=10-12. **(C)** Graphical presentation of absolute CD4^+^ and CD8^+^ cell numbers in pLN of the indicated mice. Mean and SD are shown; n=9-11. **(D)** Dot plots of CD3^+^ T cells vs. CD19^+^ B cells in pLN of Atg5^fl/fl^ and Atg5^ΔOX40^ mice. **p < 0.01 and ***p <0.001.

When CD4^+^ and CD8^+^ T cell populations were analyzed, Atg5^ΔOX40^ mice exhibited a reduction in the frequencies of both subsets (**Figures 3A, B**). However, the absolute numbers of helper and cytotoxic T cells were not significantly altered, although there was a slight tendency towards an enlarged T cell compartment (**Figure 3C**). This suggests that other cell populations were expanded in Atg5^ΔOX40^ mice, which were found to be mostly CD19^+^ B cells (**Figure 3D**). In summary, Atg5^ΔOX40^ mice exhibit normal T cell development but increased cellularity within peripheral lymph nodes, suggesting a lower threshold for the activation of the immune system.

### 3.3 Peripheral CD4^+^ T cells of Atg5^ΔOX40^ mice have an activation defect

**Figure 4:**
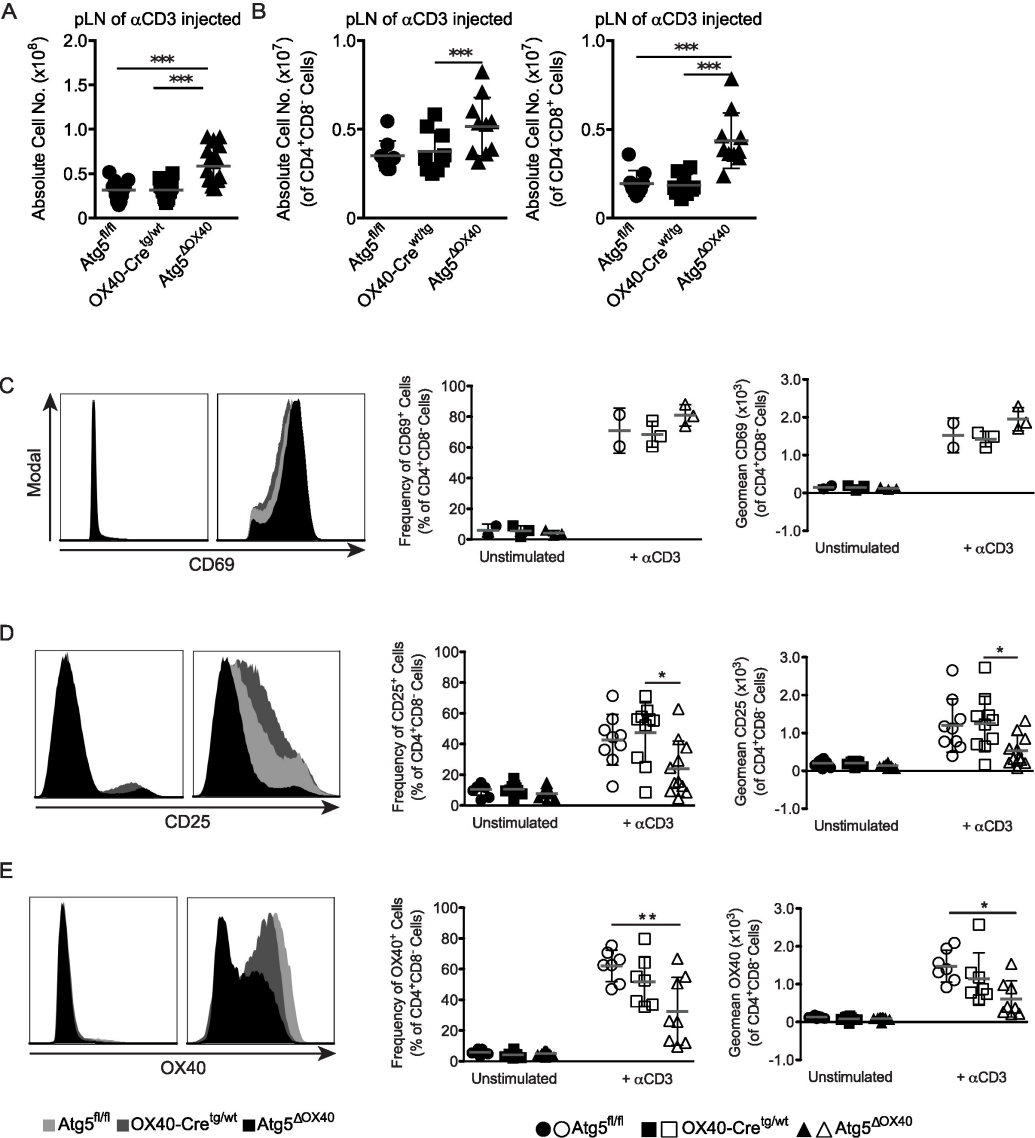
Lymphadenopathy and activation defect in peripheral T cells of Atg5^ΔOX40^ mice. **(A)** Absolute cell numbers of pLN from anti-CD3 injected mice. Mean and SD are indicated; n=12-16. **(B)** Absolute cell numbers of CD4^+^ cells and CD8^+^ cells from pLN of anti-CD3 injected mice. Mean and SD are shown; n=12-16. **(C)** Histograms of CD69 expression by CD4^+^ T cells in pLN, as well as graphs showing the corresponding frequency and geomean. Mean and SD are shown; n=2-3. **(D)** Histograms of CD25 expression by CD4^+^ T cells in pLN, as well as graphs showing the corresponding frequency and geomean. Mean and SD are shown; n=9-11. **(E)** Histograms of OX40 expression by CD4^+^ T cells in pLN, as well as graphs showing the corresponding frequency and geomean. Mean and SD are shown; n=7-9. *p < 0.05; **p < 0.01 and ***p < 0.001.

The enhanced cellularity described above might result from enhanced activation of effector cells or impaired regulatory mechanisms. In that respect, autophagy has on the one hand been connected to activation of T cells (34), but has also been shown to down-modulate TCR-induced signaling (35). To dissect between these two possibilities, we injected anti-CD3 antibodies into Atg5^ΔOX40^ and control mice to activate T cells *in vivo*. We observed an increased cellularity in secondary lymphoid organs of Atg5^ΔOX40^ mice, which was most prominent in pLN (**Figure 4A**). Accordingly, the absolute numbers of CD4^+^ and CD8^+^ T cells were increased in Atg5^ΔOX40^ mice upon anti-CD3 injection (**Figure 4B**). Thus, autophagy deficiency in CD4^+^ T cells appears not to affect T cell proliferation in general.

Subsequently, we investigated the activation status of CD4^+^ T cells by analyzing the expression of the activation markers CD69, CD25, and OX40 in mice injected with anti-CD3 or left untreated. T cell activation was not impaired in general since CD69 expression was normal (**Figure 4C**). In contrast, the late activation markers CD25 and OX40 exhibited impaired induction in CD4^+^ T cells of stimulated Atg5^ΔOX40^ mice (**Figures 4D, E**). Although OX40 expression by CD4^+^ T cells was reduced in OX40-Cre^tg/wt^ mice compared to Atg5^fl/fl^ mice due to the knock-in of *Cre* into the *Tnfrsf4* locus, OX40 induction was even further reduced in Atg5^ΔOX40^ mice (**Figure 4E**).

Taken together, although the immune system appears to be activated in Atg5^ΔOX40^ mice, ATG5 deficiency in T helper cells impairs CD4^+^ T cell activation. Therefore, the increased cellularity in the secondary lymphoid organs alludes to an altered threshold for immune cell activation, potentially due to deficient Treg suppression.

### 3.4 Loss of autophagy in Treg cells of Atg5^ΔOX40^ mice causes reduced Treg cell numbers

**Figure 5:**
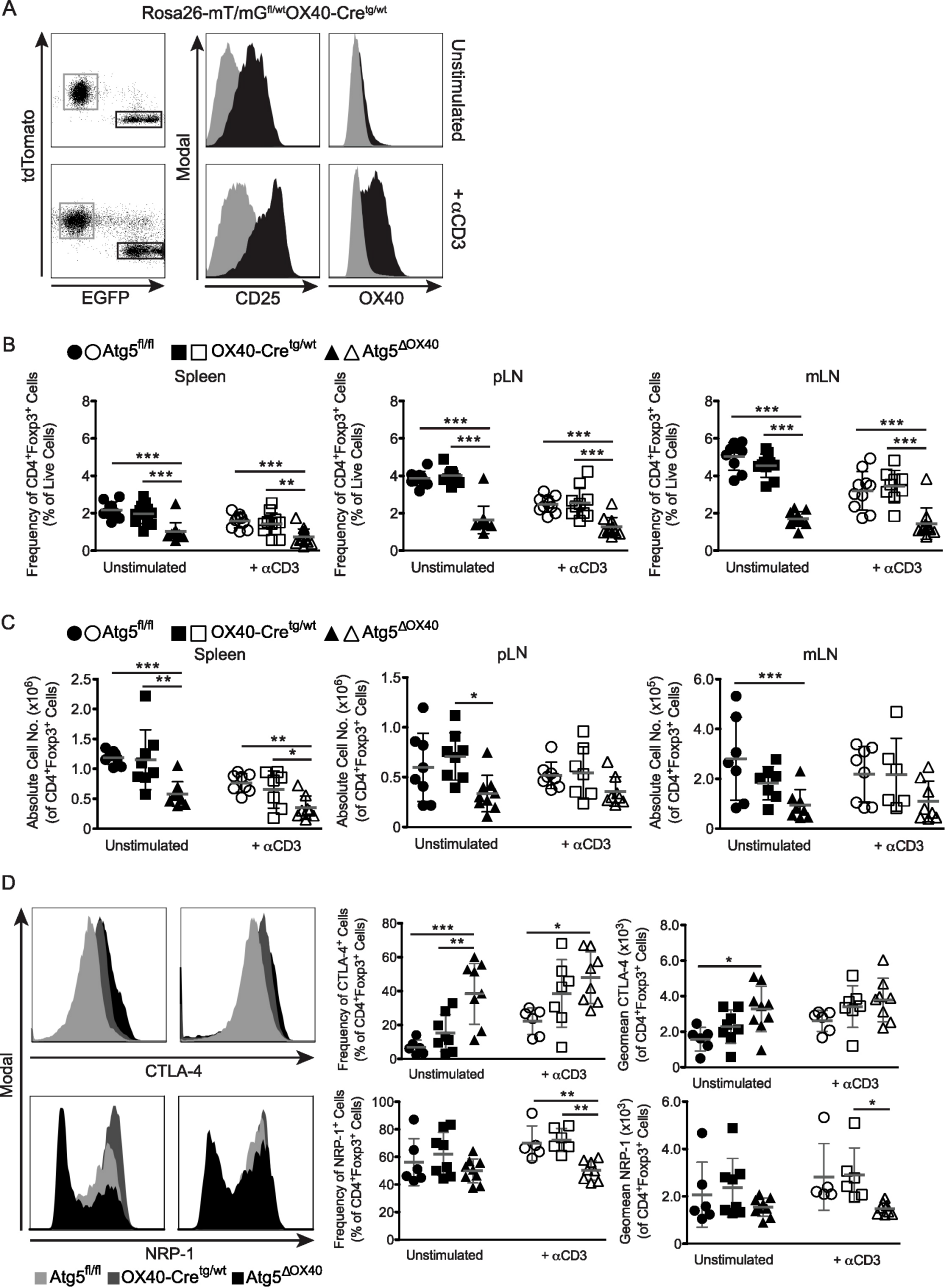
Effect of autophagy-deficiency on Treg cells in Atg5^ΔOX40^ mice. **(A)** CD25 and OX40 expression within EGFP^-^ (grey) and EGFP^+^ (black) cells from peripheral lymph nodes of Rosa26-mT/mG^fl/wt^OX40-Cre^tg/wt^ mice stimulated with anti-CD3 or left untreated. **(B)** Frequencies and **(C)** absolute cell numbers of CD4^+^Foxp3^+^ Treg cells in the indicated organs from unstimulated and anti-CD3 stimulated mice. Mean and SD are shown; **(A)** n=9–13 and **(B)** n=6-9. Statistical analysis was performed with the one-way ANOVA and Tukey’s multiple comparison tests. **(D)** Histograms and graphs depicting CTLA-4- and Nrp-1-expression by Treg cells. Unstimulated mice are shown in the left panels and anti-CD3 treated animals are depicted in the right panels. Mean and SD are shown; CTLA-4: n=6-9, Nrp-1: n=5-9. *p < 0.05; **p < 0.01 and ***p < 0.001.

Treg cells are an essential part of peripheral tolerance in the immune system. We had a closer look again at the Rosa26-mT/mG^fl/wt^OX40-Cre^tg/wt^ mice and compared the EGFP-positive with the EGFP-negative cell population from pLN. Interestingly, the former had a much higher expression of CD25 both in unstimulated and anti-CD3 stimulated mice and a higher expression of OX40 in stimulated mice, which is consistent with a Treg cell phenotype (**Figure 5A**). Indeed, most CD4^+^Foxp3^+^ Treg cells of Rosa26-mT/mG^fl/wt^OX40-Cre^tg/wt^ mice were EGFP ^+^ suggesting that OX40-dependent loss of autophagy affects especially Treg cells (**Figure 1C**). CD4^+^Foxp3^+^ Treg frequencies and numbers were reduced in peripheral lymphoid organs of both unstimulated and stimulated Atg5^ΔOX40^ mice (**Figures 5B, C**). Although several OX40 knockout studies have shown that OX40 expression is crucial for Treg development and function and that these mice have severely reduced mature Treg numbers (36–38), the effect seen in Atg5^ΔOX40^ mice was independent of reduced OX40 expression, since OX40-Cre^tg/wt^ mice did not show reduced Treg numbers in peripheral lymphoid organs (**Figures 5B, C**). Furthermore, when investigating functional Treg markers, the percentage of CTLA-4-expressing Treg cells was increased in peripheral lymphoid organs of Atg5^ΔOX40^ mice (**Figure 5D**). Analyzing additional Treg markers, we found that Nrp-1 expression was reduced in Treg cells from peripheral lymphoid organs of unstimulated and especially of stimulated Atg5^ΔOX40^ mice compared to Treg cells from control mice (**Figure 5D**).

### 3.5 Atg5-deficient OX40-positive T cells are susceptible to AICD and have suppressive activity *in vitro*

**Figure 6:**
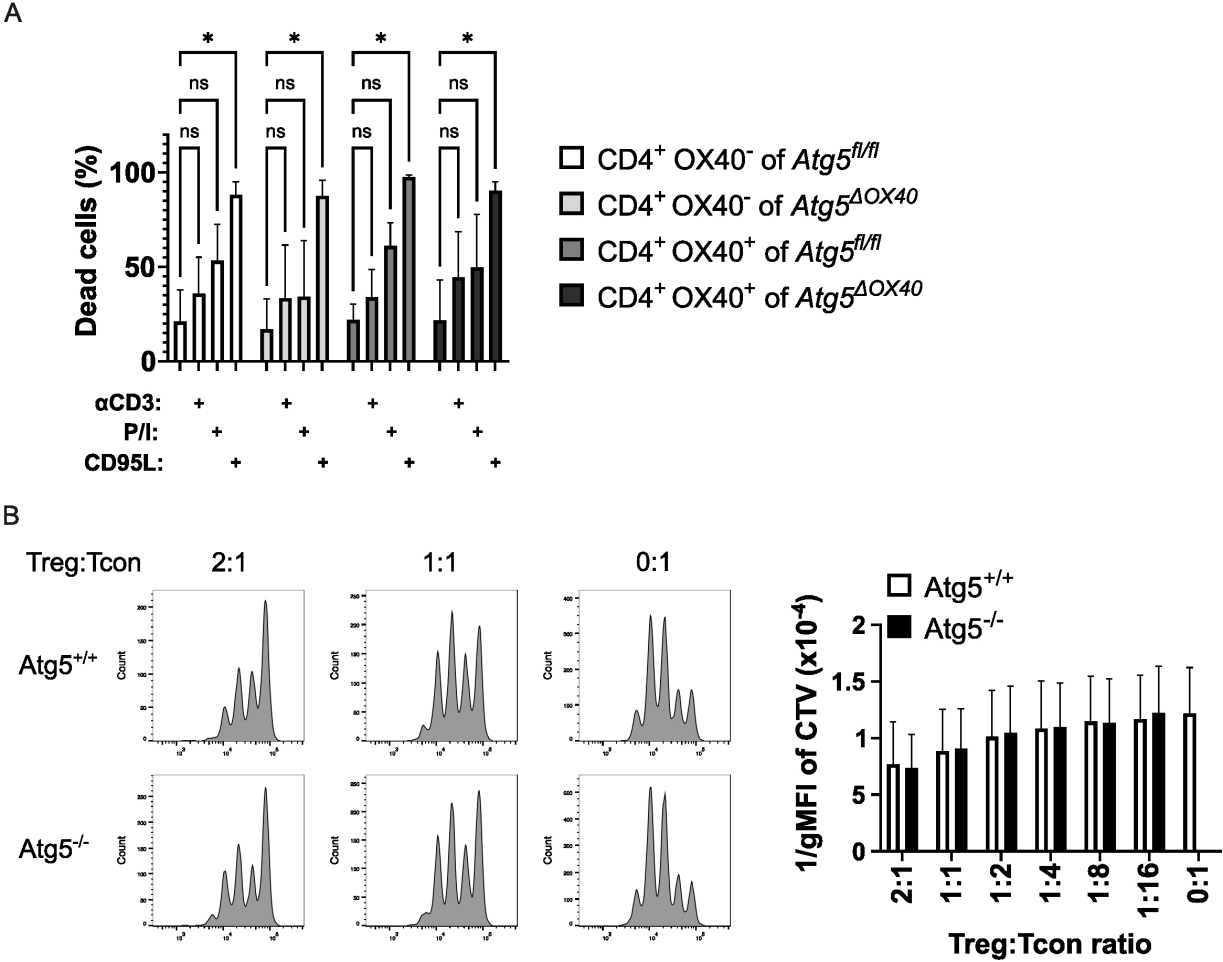
Unaltered T cell death and Treg suppressive capacity. **(A)** CD4+ T cells were activated with concanavalin A and IL-2 for 3 days and subsequently stimulated with anti-CD3, PMA/ionomycin, or CD95L. Cell death was measured with 7AAD and flow cytometry. The data shown is from three independent experiments. The two-way ANOVA Dunnett´s multiple comparison test was used for statistical analysis. **(B)** *In vitro* suppression assay comparing Treg cells from Atg5^ΔOX40^ and control mice. Wild-type conventional T cells were labeled with cell trace violet (CTV) and incubated at the indicated ratios with Treg cells of the respective genotype. Cells were analyzed by flow cytometry. Representative dot plots are shown on the left; the right panel shows the summary of three independent experiments. *p < 0.05.

Atg5 has been connected to the regulation of life and death of T cells (20, 22, 34). Therefore, we tested susceptibility to activation-induced cell death (AICD) in OX40-negative and OX40-positive T cells. To this end, CD4+ T cells were purified, activated *in vitro* and subsequently, after a short culture period, stimulated with anti-CD3, PMA plus ionomycin, or recombinant CD95L to induce cell death. While CD95L was the most potent death stimulus, no significant differences between OX40-negative and OX40-positive T cells and the two genotypes were observed (**Figure 6A**). We conclude that differences in cell death susceptibility did not account for the disturbed immune homeostasis in Atg5^ΔOX40^ mice.

Next, we tested the suppressive capacity of Treg cells from Atg5^ΔOX40^ and control mice. Conventional wild-type T cells were labeled with cell trace violet, activated via CD3 plus CD28, and incubated with Treg cells of conditional knockout or control mice. No differences in Treg suppressive activity could be detected in this *in vitro* setting (**Figure 6B**). Since Treg activity might be different *in vitro* versus *in vivo*, we went on to test Atg5-deficient Treg cells in Atg5^ΔOX40^ mice *in vivo*.

### 3.6 Loss of autophagy in Treg cells of Atg5^ΔOX40^ mice improves anti-tumor responses

**Figure 7:**
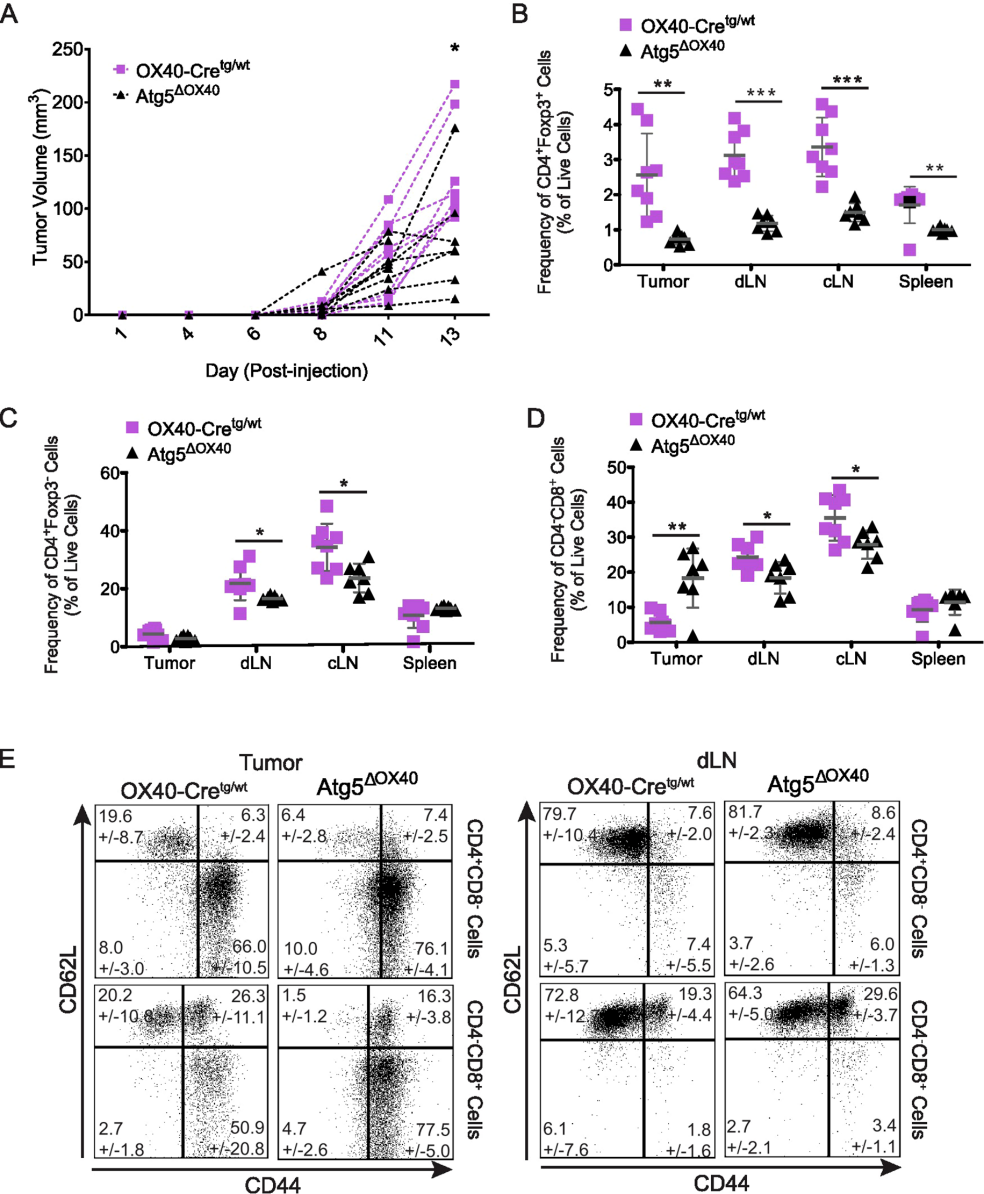
Enhanced anti-tumor response in Atg5^ΔOX40^ mice. **(A)** B16 tumor growth curves in individual mice. Atg5^ΔOX40^ mice are indicated as black triangles and OX40-Cre control mice are depicted as violet squares. **(B-D)** Frequency of CD4^+^Foxp3^+^ **(B)** and CD4^+^Foxp3^-^ **(C)** and CD4^-^CD8^+^ **(D)** cell populations in the tumor, draining lymph nodes (dLN), control lymph node (cLN) and spleen. **(E)** Dot plots of CD62L and CD44 expressing CD4^+^Foxp3^-^ and CD4^+^Foxp3^+^ cell populations from the tumor and dLN, mean frequency +/- SD are shown. One of two experiments is depicted where n=7-8. *p < 0.05; **p < 0.01 and ***p < 0.001.

Nrp-1 has been described as a marker for thymus-derived Treg cells (39, 40), but is also known to play a role in the migration of Treg cells to VEGF-expressing tumors, and this results in increased tumor growth due to Treg cells suppressing anti-tumoral responses (12). When injecting Atg5^ΔOX40^ and control mice with B16 tumor cells, tumor growth was significantly delayed in Atg5^ΔOX40^ mice (**Figure 7A**). CD4^+^Foxp3^+^ Treg cell frequencies were substantially reduced in all peripheral lymphoid organs analyzed as well as in the tumor (**Figure 7B**), whereas CD4^+^Foxp3^-^ conventional T cell frequencies were only slightly reduced in draining (dLN) and control lymph nodes (cLN) (**Figure 7C**). Furthermore, an increased frequency of intratumoral CD8^+^ cytotoxic T cells was seen in Atg5^ΔOX40^ compared to control mice (**Figure 7D**), similar to what has been reported for mice in which Nrp-1 expression was deleted (12). In turn, the frequencies of CD8^+^ cytotoxic T cells were decreased in dLN and spleen, suggesting migration of these cells from the secondary lymphoid tissue towards the tumor. Lastly, intratumoral CD62L^+^CD44^-^ naïve CD4^+^ T cell frequencies were reduced, whereas activated CD62L^-^CD44^+^ cell frequencies were increased in these mice (**Figure 7E**). CD62L/CD44 populations of CD4^+^ T cells derived from dLN, cLN, and spleen were normal (**Figure 7E** and data not shown). CD8^+^ T cells had decreased frequencies of CD62L^+^CD44^-^ naïve cells and increased frequencies of CD62L^-^CD44^+^ activated cells (**Figure 7E**). Furthermore, whereas in dLN and cLN the CD62L^+^CD44^+^ memory cell frequencies were increased, this cell population was decreased in intratumoral CD8^+^ T cells (**Figure 7E** and data not shown). In summary, our data indicate that autophagy is a critical process for the activation of T cells and supports previous findings that have illustrated the importance of this biological process for the homeostasis and function of Treg cells.

## 4 Discussion

Although the potential roles of autophagy in the activation of T cells have been investigated, published results are not conclusive. It was shown that T cells upregulate autophagy upon activation and that autophagy degrades proteins in stimulated cells, rather than performing organelle degradation as seen in unstimulated cells, in order to aid the synthesis of products necessary for cells when activated (41, 42). Most previous *Atg*-deletion studies have used *Lck*-Cre or *Cd4*-Cre mice, in which thymocyte frequencies and numbers were perturbed due to *Atg* gene deletion in thymocytes during development, resulting in increased endoplasmatic reticulum and mitochondrial mass in naïve T cells reported by most studies (43–48). These mice also exhibited both reduced CD4^+^ and CD8^+^ T cell frequencies and numbers in the periphery. However, this reduction in peripheral T cells might be due to the impaired T cell development observed in these mice. Indeed, frequencies and absolute numbers of CD4^+^ T cells were not affected in mice, in which Atg5 deletion was driven by dLck-Cre (49), where Cre expression is driven by the distal Lck promoter that is active in peripheral T cells, but not thymocytes. In contrast, naïve and central memory CD8^+^ T cells were reduced in these mice (49). To investigate autophagy specifically in CD4^+^ T cells, we generated Atg5^ΔOX40^ mice, in which Cre expression is driven by the *Tnfrsf4* (OX40) promoter that is active selectively in activated CD4^+^ T cells (27, 32). As expected, T cell development appeared unperturbed in Atg5^ΔOX40^ mice, allowing us to address the function of autophagy in peripheral T cells in an unbiased manner.

TAX1BP1, an autophagy receptor protein involved in selective autophagy, is critical to enable the metabolic transition of activated T cells and to sustain the proliferation of these cells, providing key amino acids for mTOR activation (50). Furthermore, previous studies reported normal CD25 and CD69 expression by activated autophagy-deficient T cells, and the current view is that proliferation and survival of T cells, but not the activation process per se, is autophagy-dependent (43, 45–47). Interestingly, T cells from Atg5^ΔOX40^ mice failed to upregulate the late activation markers CD25 and OX40 upon anti-CD3 stimulation *in vivo*, while the early activation marker CD69 was normally induced upon stimulation. Since OX40/OX40L signaling supports continued proliferation and clonal expansion of CD4^+^ T cells via maintenance of anti-apoptotic proteins and ensures their effector functions (36, 38, 51–54), these results suggest that autophagy is important for sustained CD4^+^ T cell activation.

Autophagy is important for the survival of T cells, including Treg cells (20, 22, 34). One mechanism is via reducing the expression of death-promoting molecules such as caspases (20, 55). Another mechanism is via mitophagy, since autophagy-deficient T cells accumulate mitochondria, which leads to enhanced production of reactive oxygen species (56). Next to survival, autophagy regulates Treg cell stability by influencing mTORC1 signaling and c-Myc expression (20). Both affect the metabolism of the cell and switch from OXPHOS, on which Treg cells usually rely, towards aerobic glycolysis, which impairs Foxp3 expression (57, 58). While the studies discussed so far mostly used Atg5 or Atg7 deletion to impair autophagy, deletion of other core autophagy proteins such as Atg16l1, Vps34 (21, 45, 59) and novel components of the pathway such as ZFP91 and RGS1 (60, 61) support the view that autophagy – and not non-autophagy-related functions of these molecules - regulates Treg survival, stability, and function. Importantly, this connection has been demonstrated for human Treg cells as well (60, 62).

The expansion of cells in peripheral lymph nodes and the increase of B cells in the Atg5^ΔOX40^ mouse line point towards a dysregulation of the immune system. Since Atg5 has been connected to cell death regulation in T cells (20, 22, 34), impaired deletion of activated effector T cells could explain this phenotype. However, we did not detect any differences in activated T cells regardless of OX40 expression in T cells from Atg5^ΔOX40^ and control mice. This, together with the reduced cell frequencies and numbers of CD4^+^Foxp3^+^ Treg cells, suggests an insufficient capacity to maintain the immune homeostasis by Treg cells. Surprisingly, the expression of CTLA-4 by Treg cells of Atg5^ΔOX40^ mice was increased. CTLA-4/CD152 is constitutively expressed by CD4^+^CD25^high^ Treg cells and is important for their suppressive function (63, 64). Thus, Treg cells might upregulate CTLA-4 in Atg5^ΔOX40^ mice to compensate for the reduced number of Treg cells present in these mice. Several studies have demonstrated that loss of autophagy proteins such as ATG5, ATG7, or ATG16L1 in Treg cells results in impaired homeostasis and functioning of these cells (20–23). Therefore, it is most likely that the defects in autophagy - and not non-canonical functions of ATG proteins - in Treg cells are the basis of the spontaneous inflammation described in those mice. Since OX40 is expressed in Treg cells and we have detected that OX40-driven Cre expression is particularly high in CD4^+^Foxp3^+^ cells, it is plausible that the Treg cells of the Atg5^ΔOX40^ mouse line have also functional deficiencies, explaining the rise of the spontaneous phenotype of immune system activation in these mice. While we were not able to demonstrate a functional impairment of Treg cells from Atg5^ΔOX40^ mice in a conventional *in vitro* assay, the better anti-tumor response in the B16 model strongly supports the notion that autophagy competence is important for Treg function.

Nrp-1 expression was reduced by Treg cells in Atg5^ΔOX40^ mice, which led us to investigate Treg cells in a tumor setting in these mice. Using a B16 melanoma model, we observed a more active anti-tumor response by CD8^+^ T cells in Atg5^ΔOX40^ mice compared to control mice. Next to reduced Treg cell numbers, the diminished Nrp-1 expression by Treg cells could cause a deficit in the migration of Treg cells to the tumor. Even the increased CTLA-4 expression by Treg cells was not sufficient to suppress the anti-tumor response in Atg5^ΔOX40^ mice injected with B16 tumor cells. Therefore, probably due to both reduced Nrp-1 expression and reduced Treg cell numbers, tumor growth was reduced in these mice. Thus, inhibition of autophagy in T cells might be a suitable therapeutic option for cancer treatment. Here, we showed that impairing autophagy selectively in CD4^+^ T cells is sufficient to increase anti-tumor responses by CD8^+^ T cells. Furthermore, it was shown that autophagy-deficient CD8^+^ T cells adopt an effector memory T cell phenotype, resulting in anti-tumor immunity (65). Therefore, targeting an autophagy inhibitor to T cells might be a promising approach in cancer therapy.

In conclusion, our study shows the relevance of autophagy in T cell activation and makes a distinction between the importance of this biological process in mature conventional and regulatory T cells. Furthermore, it points to autophagy in regulatory T cells as a potential target for anti-tumor therapies.

## Data Availability

The raw data supporting the conclusions of this article will be made available by the authors, without undue reservation.
